# Lessons Learned From Emergency Department Fall Assessment and Prevention Programs

**DOI:** 10.7759/cureus.16526

**Published:** 2021-07-20

**Authors:** Phraewa Thatphet, Fae B Kayarian, Kei Ouchi, Teresita Hogan, John G Schumacher, Maura Kennedy, Shan W Liu

**Affiliations:** 1 Department of Emergency Medicine, Massachusetts General Hospital, Massachusetts, USA; 2 Emergency Medicine, Khon Kaen University, Khon Kaen, THA; 3 Department of Emergency Medicine, Brigham and Women's Hospital, Massachusetts, USA; 4 Department of Emergency Medicine, University of Chicago Medicine, Illinois, USA; 5 Epidemiology and Public Health, University of Maryland, Maryland, USA

**Keywords:** fall, geriatric emergency, fall assessment, fall prevention, fall program, fall implementation, geriatric fall

## Abstract

Objectives

This research describes the experiences of emergency departments (EDs) with geriatric fall programs and qualitatively synthesizes lessons learned to inform other EDs planning new fall program implementation.

Methods

By using grounded theory, we conducted semi-structured, open-ended telephone/skype interviews of emergency physicians and geriatric providers recruited from a purposeful sampling technique. The interviews were transcribed and reviewed by two investigators. The codes were generated and listed, and common concepts emerged. Lastly, the final codes were organized into concepts and themes with the aim to create a strong coding structure.

Result

The main lessons learned are: (1) understand the hospital’s existing local environment and resources, (2) utilize champions and interdisciplinary teams, (3) acknowledge that specific fall assessment tools and interventions vary widely between institutions, (4) engage in routine plan-do-study-act (PDSA) cycles to improve the quality of fall initiatives, and (5) operate under the principle that falls are a syndrome, which must be incorporated within the multifactorial medical needs of geriatric fall patients.

Conclusion

Based on the lessons learned from our ED fall implementation pioneers, implementing an effective geriatric fall protocol in an ED setting is complicated. Understanding a hospital’s resources, assigning champions, working as an interdisciplinary team, choosing proper fall assessment tools/interventions, and completing regular PDSA cycles are important lessons for ED programs planning to implement their own ED fall programs.

## Introduction

Background

Falls are the leading cause of unintentional injury and death among individuals aged 65 years and older in the United States [1]. Since 2010, the rate of falls among geriatric patients has been increasing. In 2016, 7 million falls were estimated, which accounted for an overall one-fourth of older adults in the US [2]. The number of older people with fall-related causes of death rose from approximately 8,600 people in 2000 to more than 32,000 people in 2018 [3]. In 2010 alone, falls accounted for three million emergency department (ED) visits in the United States, with geriatric patients representing 71% of all patients with fall-related presentations [4]. Any fall, especially those significant enough to result in an ED visit, adversely affects the quality of life and health outcomes of geriatric patients. Additionally, fall patients place a heavy financial burden on the United States healthcare system. Medical expenses relating to geriatric falls and fall-related injuries have been rising from $38 billion in 2013 to $50 billion in 2015 and are expected to cost $67.7 billion in 2020 [5-7]. Preventing falls in older patients not only reduces these costs but also the morbidity and mortality rates in geriatric patients.

There is a growing emphasis on the role of the ED in the management of older adult falls. Multiple studies have implemented various tools to better assess and identify geriatric patients at risk for future falls as well as the interventions to prevent future falls starting in the ED [8-12]. The Panel on Prevention of Falls in Older Persons, the American Geriatrics Society, the British Geriatrics Society [13], and the Centers for Disease Control and Prevention [14] recommend implementing a multifactorial fall risk assessment and management of geriatric patients who present with falls or with gait and balance problems. Moreover, the Geriatric Emergency Department Accreditation (GEDA) Program formally accredits EDs that implement policies to improve geriatric ED care based on the Geriatric Emergency Department Guidelines [15,16]. GEDA offers three levels of accreditation, with varying intensity of requirements. Fall risk assessment and prevention is one of the geriatric issues in GEDA’s accreditation criteria that EDs can focus on to improve geriatric care. However, initiatives to reduce fall rates among ED geriatric fall patients have produced mixed outcomes and many ED healthcare providers and administrators have reported initial difficulty implementing and adhering to the guidelines [17,18].

Importance

While various studies of fall prevention programs exist, information about the implementation of these fall prevention program is still limited and difficult to assess [8-12]. To date, there has not been a study to explore the lessons learned from implementing ED fall assessment and prevention programs.

Goals of this investigation

The purpose of this research study is to describe the experiences of EDs that have developed geriatric fall programs and qualitatively synthesize the lessons learned to inform other EDs planning to implement their own fall programs.

## Materials and methods

Study designs

Between August 2018 and July 2019, by using grounded theory [19], we conducted semi-structured, open-ended telephone/skype interviews with three physicians, two ED nurses, and one program coordinator who implemented and managed a fall program in their given EDs. The primary aim of the interview was to explore how these programs designed and implemented their fall programs. This study was exempted by our institutional review board.

Population and setting

We used a purposeful sampling technique to enroll three physicians, two ED nurses, and one program coordinator who played a major role in setting each institution’s protocols as the key informants (KIs) from five hospitals EDs located in the US and Canada. These ED fall programs were among the first to design and implement fall programs in ED settings. The hospital sizes ranged from 400 to 1,000 beds and included academic and non-academic institutions.

Study protocol 

Interviews were scheduled and conducted by a phone call or Skype video call. The interview instrument was created by SL, JS, and TH, who are experts in fall prevention protocols and/or qualitative methods. The instruments were piloted on two physicians with falls program implementation experience and refined to ensure they covered the scope and context of our primary research aim. Interviews were conducted by the PI (SL) and co-investigator (KO), who are ED physicians with qualitative research training. The call recordings were transcribed by SL and KO (interview instrument in Appendix).

Data analysis 

After the data collection period, the transcripts from each interview were independently reviewed by two of the investigators (SL, PT) to gain an understanding of the context, key concepts, and scope. The investigators (SL, PT) independently generated the codes [20] following established qualitative research methods based on grounded theory [19], discussed common concepts, and generated a final code list. From two sets of independent codes by two investigators, by using units of a specific text, a kappa score of 0.91 was generated to assess inter-rater agreement between the independently coded transcripts of a subset of interviews. The two reviewers (SL, PT) discussed and assigned the final codes through negotiated consensus. Finally, the codes were organized into concepts and themes to create a strong coding structure that corresponded to an article by Ranney et al. for Interview-based Qualitative Research in Emergency Care [20].

## Results

Three physicians, two ED nurses, and one program coordinator from five hospitals were interviewed as KIs. The brief data of each hospital will be referred to as sites (A-E; Table 1).

**Table 1:** Description of the key informants work settings

Site	Hospital location	Setting of hospital
A	Illinois, USA	Urban teaching hospital (811 beds) with 96,000+ ER visits per year
B	Ontario, Canada	Urban teaching hospital (442 beds) with 45,000+ ER visits per year
C	Wisconsin, USA	Community healthcare system including 15 hospitals
D	Massachusetts, USA	Urban teaching hospital (673 beds) with 55,000 + ER visits per year
E	New Jersey, USA	Urban hospital (nearly 1,000 beds)

Overall, there was a strong consensus among KI’s that falls among older adults remain a challenge in emergency medicine. The majority of KI’s interviewed endorsed the idea that improved fall assessments and prevention interventions, enforced at both the individual and departmental level, must be appropriately applied to improve the outcomes of geriatric fall patients. The following section describes the noteworthy lessons expressed by our KI’s (Table 2).

**Table 2:** Summary of the lessons learned from key informants PDSA: plan-do-study-act.

Theme	Lesson learned
1	Start with the hospital’s existing local environments and resources
2	Champions and interdisciplinary teams are fundamental
3	Specific falls assessment tools and interventions varied widely
4	PDSA cycles are used for quality improvement of fall initiatives
5	Fall is a syndrome

Theme 1: Start with the hospital’s existing local environments and resources

Each surveyed site has a diverse range of environmental conditions and institutional resources. Many KI’s expressed the importance of developing a geriatric fall protocol based on the resources available at a given ED. Available personnel resources vary widely, in fact, some institutions have personnel specifically dedicated to the clinical care and management of geriatric patients. For example, some sites staffed geriatric EM physicians, geriatric EM nurse practitioners (GEM NP), geriatric nurses, case managers, and palliative care nurses. Overall, these clinical team members were integrated into the ED staff to enhance care for the specific needs and challenges of geriatric patients. This can be seen from this quote from our KI.

“…there is a geriatric emergency medicine nurse practitioner (GEM NP) who sees all geriatric patients independently or in conjunction with the ED provider.” 

Additionally, some sites expanded their clinical resources by collaborating with outside community healthcare centers to enhance care for geriatric fall patients prior to or following ED hospital visits. According to several KIs, reminding providers about the fall pathway or protocol in resident didactics and faculty meetings helped encourage providers to participate in the program in academic hospitals.

Beyond using specific staff resources, some sites are able to use the electronic medical records (EMR) system to improve care for geriatric fall patients.

“[Patients] would be flagged…if [fall was] in the chief complaint or triage note free text. Then the computer would flag the fall pathway and the pathway would pop up and you would approve or not approve the pathway.”

A quote from one surveyed site utilizes an EMR system that alerts the supervising ED physician whenever a triage nurse initially encounters a geriatric fall patient.

ED fall screening tests are often used to identify high-risk geriatric fall patients. The majority of sites reported screening these patients if their chief complaint was associated with a fall. Some sites also use screening tools available in their EMR system to identify patients as at-risk for future falls.

Theme 2: Champions and interdisciplinary teams are fundamental

Because geriatric fall patients require highly coordinated care, it is important to foster interdisciplinary teamwork between physicians, GEM NPs, geriatric/palliative nurses, nurses, physical therapists (PTs), occupational therapists (OTs), nurse aides, case managers, as well as community primary care providers. This need for interdisciplinary efforts is best echoed by one KI’s feedback, which states:

“…once the person is cleared by the provider such that they are stable for weight-bearing, then they get orthostatic vital signs, BP, pulse, and the Timed Up and Go (TUG) test. If the patient has a TUG >14 seconds, then the nursing staff determines if the provider wishes to obtain a PT consult. If during day time hours, then the unit coordinator [calls PT], then PT will assess the patient. If at night, the health unit coordinator leaves a message with PT, whether they are admitted or sent back home for outpatient. It is very much identify, screen, and assess with the intervention being PT doing a further assessment. Orthostatics were done by the EM tech which is the nurse’s aide, assistant to nursing staff. The TUG was done by nursing staff and entered into the records…”

Another KI also pointed out the importance of interdisciplinary teamwork.

“If the provider/NP feels the need, PT/OT is consulted and completes the more comprehensive assessment in the ED.”

Working within an interdisciplinary team model requires open and dynamic communication between each ED staff member. Furthermore, several KI’s recommended creating a core team leading the ED fall program. The core team champion would be responsible for leading a small, nuclear group of clinical and support staff. In the long run, this ED geriatric falls champion would educate and encourage other ED team members to develop and implement initiatives aimed to improve care for geriatric fall patients.

“There are four to fivr champions who did the protocol. When all the kinks were worked out, they would train staff and would not implement [the program] fully until the kinks were worked out. We started initially with a small core team.”

To have a small group of ED providers (the champions) who are dedicated to the fall program and can enhance and maintain the ED fall protocol in the hospital seemed better than trying to use all providers at the same time. As one of the KI states:

“[The falls program] may be better if it has a smaller group of providers in a smaller facility. In the large group, it is hard to get 100 nurses, 70 physicians for a very large number of patients [to do the falls evaluation]. The large academic centers likely have a dedicated nurse or fall person who does the fall evaluation.”

The champions can be a group of people who were assigned to play a major role in taking care of older patients. While one ED site has no geriatric fall protocols established, others follow a standard fall protocol. One KI described that, while his ED does not have a concrete geriatric fall protocol established, his ED has staffed a geriatric EM nurse practitioner (GEM NP) responsible for every geriatric patient treated. The GEM NP within this academic training center is also responsible for instructing ED interns, residents, and physicians about the specific clinical needs and challenges of this vulnerable patient population. Moreover, this KI reported that his site had established a robust PT/OT consultation pathway and outpatient geriatric care coordinator pathway, facilitating PT/OT evaluation of geriatric fall patients. Given the fact that the champions might not be able to cover the ED around the clock, KIs suggested having the champions cover the hours that are likely to have more geriatric patients, for example, weekday morning shifts.

Theme 3: Specific falls assessment tools and interventions varied widely

Given the lack of any one best ED fall screening and assessment tool, multiple ED fall screening tool options exist, e.g., the TUG test [9], Morse Falls Scale (MFS) [21], St. Thomas’ Risk Assessment Tool in Falling Elderly Inpatients (STRATIFY), Falls Risk Assessment Scale for the Elderly (FRASE), and various Falls Risk Assessment Tool (FRAT). In our study, the choice of screening and assessment tools depended on each site’s familiarity and ease of use with the various tools. Most ED sites in our study utilize geriatric fall protocols to screen older patients who present with high fall risk. For example, two KI’s use the Morse Fall Risk score >44 [21] to diagnose geriatric patients with high fall risk and provide them appropriate fall prevention protocol. For instance, these high-risk patients are identified in their beds in the emergency department with a yellow pair of socks, a yellow blanket, a wrist band, and a sign labeled “fall risk.” The staff has also been instructed to keep these patients’ room curtains open, in an effort to better visually monitor the patients. Another surveyed site used a geriatric fall protocol from an outside hospital which included surrounding primary care health providers to establish the protocol so that the ED could extend its resources and clinical services.

The use of different fall screening tools may introduce variability in the care provided to geriatric ED patients. There is also the use of various tools to determine risk factors for falls, for example, cognitive impairment, dependent on daily living activities, and depression. The most commonly used falls risk factors screening test was the TUG test. KI’s from one site said they chose the TUG because it *“fit with what ED providers already say is a road test”* and it is part of the Stopping Elderly Accidents, Deaths, and Injuries (STEADI) program.

Theme 4: PDSA cycles are used for quality improvement of falls initiatives 

While the type of ED geriatric fall protocols varied from institution to institution, adaptation through multiple plan-do-study-act (PDSA) cycles was important, as one KI recommended:

“…do small PDSA cycles… think carefully about how the falls protocol fits within the larger falls prevention project. The emergency department falls protocol is like the starting point for this particular quality improvement [project].”

The process of implementing a geriatric fall protocol is often complicated and difficult to initiate in an ED setting. To facilitate and drive this process, most sites reported scheduling regular ED staff meetings and routinely completing PDSA cycles. This practice helped team members to better identify problems specific to geriatric fall patients and develop the most suitable protocols for their site. These ED staff meetings included ED providers, as well as clinical staff from other departments (neurology, neurosurgery, trauma, general surgery, etc.), institutional faculty members, and program leaders. One KI shared the example of the PDSA Cycles.

Plan: - To implement TUG testing from a triage chair on all fall patients who walk into the ED and sit in a triage chair

- Place a 10-foot-long red 3-inch tape line on the floor to the right of the triage chair. The tape will have a perpendicular line at the end extending for 2 feet in a “T” to indicate end.

- Triage RN/tech to instruct patient on standard TUG instructions and start timer on the “go command”

- Triage RN/tech to document TUG time

Do: Triage personnel follow above protocol for one-month period

Study: - Numbers of fall patients who walked to triage chair

- Ability to implement in at subgroup: number of patients

- TUG score of those tested

Act: How will we know the change is an improvement? Improvement will be considered if we see a rise in total number of older adult fall patients who get a TUG test. If numbers improve, we will seek to make this a routine part of the triage process.

Remaining issues: Unable to establish true numbers of potential TUG eligible patients:

 1. As discussed in PDSA cycle #3-

 There is no verification of total patients who were asked fall triage questions

 There is no verification of positive/negative responses to fall triage questions

 2. There is no verification of number of fallen elders who walked into ED and sat in triage.

Conclusion: Overall increase in numbers is substantial and protocol remains in place.

Theme 5: Fall is a syndrome

A major lesson is that geriatric falls are a complicated syndrome. As one KI described: 

“One of the challenges with fall pathway was the complexity… Falls are more of the syndrome.”

Given the reason patients' fall is multifactorial, it is difficult to detect, assess, manage, and prevent under a single standard protocol [22,23]. Unlike pulmonary embolism, which affects the respiratory system and has a screening blood test and radiographic test and treatment, falls are much more complicated. Falls are caused by many factors, and consequently, treatments need to be directed toward those factors [22,23]. To address the complicated nature of geriatric ED falls, each institution should individualize its own protocol by assessing its own resources, using a multidisciplinary team with a champion who decides which fall tools are most appropriate, and reassesses the efficacy of protocol by regular PDSA evaluations.

Although geriatric falls are complicated and difficult to prevent and assess, these lessons learned from our surveyed KI’s will help to improve the management of falls and ensure better care for geriatric patients in the ED.

## Discussion

Our study found that given that falls represent a complicated syndrome, ED fall programs are varied. However, the overall lessons learned from the pioneering fall programs include examining the hospital’s existing local environment and resources, setting up the champions, and interdisciplinary teams, selecting specific fall assessment tools, and ensuring regular PDSA cycles can help to successfully establish ED fall programs. 

Our findings are consistent with previous studies. In terms of champions, Koh et al. [18] recommended assigning a dedicated member of the nursing staff to be a change champion. The champion would reinforce and encourage other providers to adhere to the guidelines and also assign a “fall nurse specialist” to screen patients, alert other healthcare providers, and educate the patient and caregiver before discharge. They also recommended undergoing frequent audits to assess the quality of compliance to fall risk assessment tools. Tate [24] recommended assigning a nurse specialist to serve as a coach for the ED care team of Mobile Acute Care of the Elderly (MACE). This MACE team enhances the care for geriatric patients in an acute/ED setting and could include a geriatrician-hospitalist, a geriatric medicine fellow, a social worker, and a nurse specialist for Geriatric Care Management.

Not surprisingly, many fall programs utilize PDSA cycles. Taylor et al. [25] suggested that the PDSA cycle can help to improve the effectiveness of the development progression. The PDSA cycle is the most commonly used approach for rapid cycle improvement in healthcare through a method coined “trial-and-learning” [26]. Our KI’s discussed the importance of PDSA cycles. This is similar to studies indicating completing PDSA cycles serves to improve healthcare management in situations ranging from reducing ED waiting times to increasing patient satisfaction in primary care [27].

Our study also found that interdisciplinary teamwork was a key component in fall programs. The Health in Aging Workgroup on Multidisciplinary Competencies in Geriatrics stated that communicating and collaborating with multiple healthcare professionals who work with geriatric populations will help to improve better health outcomes for geriatric fall patients in the ED [28]. A previous study mentioned the impact of the caregiver and/or the patient’s family preventing falls but none of our KI mentioned that [29].

Overall, fall is a syndrome, which makes it more challenging to screen and prevent than other conditions, given their multiple risk factors and interventions. There are also various risk factors that affect outcome severity status post-fall. Falls and their medical consequences can affect a patient’s physical health, psychological well-being, and his/her ability to complete activities of daily living (ADLs), occupational health, economic and financial security [30]. Therefore, it is crucial to assess geriatric fall patients and prevent future falls using a multidisciplinary approach. For instance, the nurse or nurse’s aide should be responsible for measuring a geriatric fall patient’s vital signs and taking a brief history report; the physician should manage the patient’s diagnosis and treatment; the PT should help patients maintain their physical activity; the OT will allow patients to get back to their jobs; the case manager and social worker should help to discharge and transfer patients to their primary care health provider. This process is extensive and is often time-consuming, as it involves multiple systems and specialties to complete the evaluation and subsequent treatment of geriatric fall patients. There were previous studies that tailored their preventions program by each patient’s conditions and risk factors and they were more effective than adopting one program to all patients [10].

Based on the lessons learned from our ED fall implementation pioneers, implementing an effective geriatric fall protocol in an ED setting is complicated. Understanding a hospital’s resources, assigning champions, working as an interdisciplinary team, choosing proper fall assessment tools/interventions, and completing regular PDSA cycles are important lessons for ED programs planning to implement their own ED fall programs. Future studies should analyze the impact of such fall programs on fall reduction.

Limitations and future direction

The use of purposeful sampling to include EDs with a history of long-term success restricted our samples to urban teaching hospitals. Therefore, these learning points from institutions with long-term success may not directly apply to institutions in rural settings that usually have more limited personnel resources or the institution in other countries with a different system of care. Future research should aim to focus on diverse ED settings. The small number of the KI made the thematic saturation difficult to reach. Furthermore, KIs may not have shared some challenges given the potential for social desirability bias. There may have been other fall programs that we did not know which may have very different viewpoints and ED fall program components. Also, there may also have been hospitals that tried and fail to develop a fall program that would provide an interesting counterpoint view. However, such sites are difficult to find, as that information is not typically advertised or published.

## Conclusions

Based on the lessons learned from our ED fall implementation pioneers, implementing an effective geriatric fall protocol in an ED setting is complicated. Understanding a hospital’s resources, assigning champions, working as an interdisciplinary team, choosing proper fall assessment tools/interventions, and completing regular PDSA cycles are important lessons for ED programs planning to implement their own ED fall programs. Future studies should analyze the impact of such fall programs on fall reduction.

## Appendices

Interview instrument

1)  How to Identify who is a faller? 

2)  How to design a specific ED fall protocol? Which guideline(s)/algorithm(s) used? Who implements the guideline and each component? How did you implement or get support from stakeholders?

3)  Which tests do you use and which patients should undergo these specific tests?

4)  Which technology do you use, e.g., for tracking, follow-up?

5)  How do you involve clinicians and motivate clinicians to adopt? 

6)  What are the challenges you have faced?

7)  Any other advice for other programs that have implemented falls programs?
